# QTL Mapping of Kernel Traits and Validation of a Major QTL for Kernel Length-Width Ratio Using SNP and Bulked Segregant Analysis in Wheat

**DOI:** 10.1038/s41598-019-56979-7

**Published:** 2020-01-08

**Authors:** Fang Xin, Ting Zhu, Shuwei Wei, Yucui Han, Yue Zhao, Dazhong Zhang, Lingjian Ma, Qin Ding

**Affiliations:** 1 College of Agronomy, Northwest A&F University, Yangling, 712100 Shaanxi China; 2 College of Horticulture, Northwest A&F University, Yangling, 712100 Shaanxi China

**Keywords:** Agricultural genetics, Plant breeding, PCR-based techniques, Plant molecular biology, Genetic mapping

## Abstract

One RIL population derived from the cross between Dalibao and BYL8 was used to examine the phenotypes of kernel-related traits in four different environments. Six important kernel traits, kernel length (KL), kernel width (KW), kernel perimeter (KP), kernel area (KA), kernel length/width ratio (KLW), and thousand-kernel weight (TKW) were evaluated in Yangling, Shaanxi Province, China (2016 and 2017), Nanyang, Henan Province, China (2017) and Suqian, Jiangsu Province, China (2017). A genetic linkage map was constructed using 205 SSR markers, and a total of 21 significant QTLs for KL, KW, KP, KA, KLW and TKW were located on 10 of the 21 wheat chromosomes, including 1A, 1B, 2A, 2B, 2D, 3D, 4D, 5A, 5B, and 7D, with a single QTL in different environments explaining 3.495–30.130% of the phenotypic variation. There were four loci for KLW, five for KA, five for KL, three for KP, two for KW, and two for TKW among the detected QTLs. We used BSA + 660 K gene chip technology to reveal the positions of major novel QTLs for KLW. A total of 670 out of 5285 polymorphic SNPs were detected on chromosome 2A. The SNPs in 2A are most likely related to the major QTL, and there may be minor QTLs on 5B, 7A, 3A and 4B. SSR markers were developed to verify the chromosome region associated with KLW. A linkage map was constructed with 7 SSR markers, and a major effect QTL was identified within a 21.55 cM interval, corresponding to a physical interval of 10.8 Mb in the Chinese Spring RefSeq v1.0 sequence. This study can provide useful information for subsequent construction of fine mapping and marker-assisted selection breeding.

## Introduction

Wheat (*Triticum aestivum* L.) is one of the most widely grown crops worldwide and a major contributor to the global diet. The most important goal of wheat breeding is high yield and quality, which is also the basis of meeting the needs of rapid population growth and improving living standards. It is of great theoretical and practical significance to understand the genetic traits related to characteristics of the wheat kernel. Kernel traits, including kernel length (KL), kernel width (KW), kernel perimeter (KP), kernel area (KA), kernel length/width ratio (KLW), and thousand-kernel weight (TKW), are important indices of wheat quality and yield. Among these traits, TKW is one of the important yield components in wheat. It can be affected by KL and KW^1^. KLW is closely related to kernel milling quality and commercial value^2^, where the large, round and full filled kernels have higher flour yield than the small, thin and flat filled kernels^3^. However, the kernel traits of wheat are easily affected by the environment, which is a typical representative of quantitative trait. The emergence of quantitative trait locus (QTL) analysis provides an effective method to analysis these complex traits^4^.Therefore, QTL mapping is considered to be the best method to study the quantitative traits of crops. At present, this method has been widely used in research on the quantitative traits of many crops.

Because of their importance, numerous studies on kernel traits have been reported. Among these, a large number of QTLs for TKW have been found on almost all chromosomes of wheat^1,2,5–9^. However, the QTLs controlling the same traits differ in number, location and effect in different studies. With the development of molecular marker technology, many studies have detected QTLs associated with other kernel traits. Previous studies have identified some QTLs related to KL and KW^1,6,8,10–14^. In addition, some QTLs for KA and KP^1,5,6,10,12,15–17^ have been found in previous studies. At present, there are relatively few QTL reports for KLW, only see Kumar^6^, Li^14^ and Kumar^15^.

In the 1990s, the first wheat genetic map was successfully constructed by using RFLP markers^18^. Subsequently, PCR-based molecular markers (RAPD, AFLP and SSR) have become the main molecular markers for the construction of genetic maps, and SSR is the most widely used marker at present. However, the genetic diversity of these markers is poor, the process is time- consuming and labor- intensive, and the constructed map density cannot meet the requirements of gene fine mapping and gene cloning. SNPs are the most prevalent form of polymorphism in any biological genome^19^. SNPs can be used to construct high-density linkage map and fine mapping of target QTLs or genes. Wang^20^ constructed a wheat integration map containing 80277 SNPs using eight DH lines and analyzed the distribution of SNP loci on the chromosomes of the 90 K gene chip. The Chinese Academy of Agricultural Sciences and Affymetrix designed the 660 K SNP array of wheat in 2016 (http://wheat.pw.usda.gov/ggpages/topics/Wheat660_SNP_array_developed_by_CAAS.pdf).Compared with the 90 K chip, the number of SNPs was significantly increased in the 660 K chip. Bulked segregant analysis (BSA) mainly selects individuals with extreme phenotypes from the offspring of parental groups to be mixed^21^. By comparing the polymorphisms between different extreme mixing pools and combining phenotypes, target genes can be located. There are many molecular markers used in gene localization combined with BSA (e.g. RAPD, AFLP, SSR), but they are still time- consuming and labor- intensive, also difficult to simultaneously perform large-scale localization screening of a large number of materials or target genes. With the development of DNA sequencing technology, SNP markers have shown advantages such as high quantity, high density, high genetic stability and easy automatic detection. SNPs are thus rapidly replacing traditional markers such as RFLP and SSR, and SNPs are now widely used in the construction of animal and plant genetic linkage maps. The BSA + gene chip method can quickly detect the genetic differences between two different phenotypes^22,23^. The SNP mutation and its chromosomal segment are more accurate than genome-wide gene mapping.

The goals of this study were (1) to identify QTLs for kernel- related traits (TKW, KA, KP, KL, KW and KLW) using recombinant inbred lines (RIL) derived from the cross Dalibao × BYL8 and (2) to use the BSA + 660 K technique to mine SNPs associated with KLW and compare the results with those using SSR markers to validate the major QTLs for KLW.

## Results

### Phenotypic variation

Experiments were conducted in four different environments. The mean, standard deviation, kurtosis, skewness, range, and the broad sense heritability were calculated for each of the phenotypes in the RIL populations. Descriptive statistics for these kernel traits are presented in Table 1. The *t*-test showed that the two parents were significantly different (*p* < 0.05) for all investigated kernel traits. For KLW, Dalibao consistently showed higher values than BYL8 in all tested environments. The TKW of BYL8 was higher than that of Dalibao in all environments except Suqian during 2017–2018. For KA and KW, BYL8 kernels were larger and wider than those from Dalibao, except at Yangling during 2017–2018. For KP and KL, Dalibao kernels were always longer than those of BYL8, except at Yangling during 2017–2018. All kernel traits had broad-sense heritability higher than 95%.

**Table 1:** Traits of kernel related traits in parents and RILs derived from Dalibao/BYL8 at four environments.

Trait	Env.	parent		T-value	RIL population					
		Dalibao	BYL8		Mean	SD	Kurtosis	Skewness	Range	CV
TKW (g)	E1	35.932	43.881	−2.993^*^	47.748	9.18	0.452	−0.791	15.515–65.881	19.225
	E2	52.523	56.389	−2.930^*^	51.664	11.335	0.484	0.799	22.738–57.541	21.94
	E3	27.833	33.791	−9.785^**^	31.504	5.426	0.483	0.213	16.961–51.564	17.222
	E4	52.232	49.521	6.120^**^	51.39	30.174	0.898	0.416	27.126–59.352	18.716
KA (mm^2^)	E1	21.039	21.205	−0.187^*^	23.253	2.544	0.276	−0.495	14.528–29.583	10.94
	E2	21.344	23.951	−3.253^*^	20.956	1.717	0.808	−0.159	13.593–26.385	8.193
	E3	18.458	19.551	1.199^*^	18.509	1.683	0.827	−0.116	12.922–23.967	9.092
	E4	22.895	20.103	1.933^*^	20.479	1.767	0.162	−0.142	14.981–25.670	8.628
KP (mm)	E1	20.789	19.176	21.913^**^	20.217	0.914	0.504	−0.227	16.991–22.891	4.521
	E2	19.746	22.498	−16.417^**^	20.038	0.902	0.8	−0.319	15.881–22.597	4.502
	E3	20.12	19.307	−1.087^*^	19.162	0.886	0.888	−0.508	15.54–21.487	4.622
	E4	21.162	18.87	4.704^**^	19.198	0.881	0.366	−0.146	16.508–22.600	4.587
KLW	E1	2.801	2.167	11.400^**^	2.222	0.197	0.159	0.756	1.868–2.854	8.884
	E2	2.344	1.955	30.558^**^	2.127	0.119	1.005	0.426	1.836–2.754	5.589
	E3	2.566	2.04	8.929^**^	2.223	0.123	0.187	0.159	1.918–2.622	5.555
	E4	2.251	1.842	5.069^**^	1.987	0.099	0.154	0.373	1.740–2.342	4.973
KL (mm)	E1	8.658	7.615	4.529^**^	8.135	0.372	0.579	−0.199	6.857–9.240	4.567
	E2	7.337	8.69	−23.887^**^	7.58	0.346	1.787	−0.461	5.726–8.802	4.571
	E3	7.724	7.069	2.417^*^	7.209	0.337	0.915	−0.439	5.726–8.181	4.678
	E4	8.037	6.836	8.048^**^	7.178	0.333	0.27	−0.223	6.081–8.113	4.636
KW (mm)	E1	3.132	3.589	−5.374^**^	3.716	0.319	0.196	−0.718	2.612–4.379	8.579
	E2	3.768	3.724	1.047^*^	3.601	0.186	0.969	−0.333	2.716–4.172	5.179
	E3	3.044	3.518	−9.680^*^	3.298	0.199	0.557	−0.071	2.568–3.960	6.039
	E4	3.607	3.738	−1.703^*^	3.643	0.185	0.523	−0.464	2.946–4.112	5.08

TKW thousand kernel weight, KA kernel area, KP kernel perimeter, KLW kernel length/width ratio, KL kernel length, KW kernel width, Hb2broad-sense heritability.

**Figure 1:**
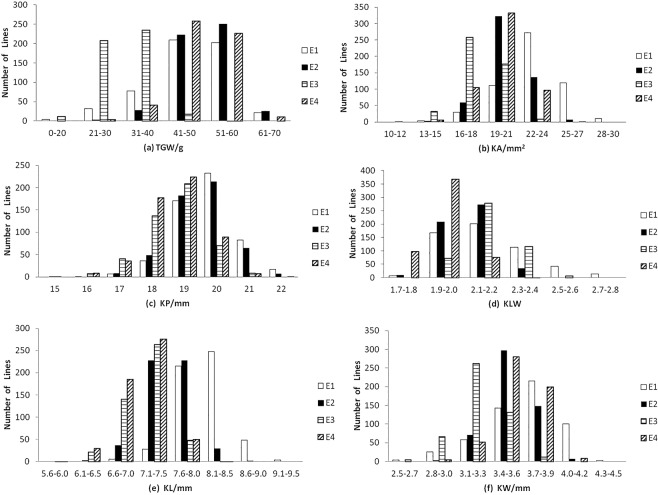
Histograms of distributions for wheat kernel related traits in Dalibao/BYL8 RIL population. *TKW* thousand kernel weight (**a**), *KA* kernel area (**b**), *KP* kernel perimeter (**c**), *KLW* kernel length/width ratio (**d**), *KL* kernel length (**e**), *KW* kernel width (**f**) *E1* Yangling 2016–2017, *E2* Yangling 2017–2018, *E3* Nanyang 2017–2018, *E4* Suqian 2017–2018.

According to the phenotypic distribution of kernel traits in parents and their RIL population, TKW, KL, KW, KA, KP, and KLW traits of wheat are easily affected by environmental conditions. The RIL population showed continuous variation in the six kernel traits, and the range of variation was large. There was obvious two-way super-parent separation (Table 2; Fig. 1). The patterns were typical of quantitative traits, because the scores of skewness and kurtosis were mostly less than 1.0.

**Table 2:** Mean squares of ANOVA and heritability for kernel traits of RILs.

Source of variation	Df	TKW	KLW	KW	KL	KA	KP
Year	3	47500.62^**^	5.48^**^	14.81^**^	83.13^**^	36.47^**^	14.66^**^
Genotype	141	363.45^**^	0.19^**^	0.44^**^	1.12^**^	1177.90^**^	273.60^**^
Genotype × Year	423	11.20^**^	0.01^**^	0.01^**^	<0.01^**^	1.53^**^	0.49^**^
Error	1136	0.02	<0.01	<0.01	<0.01	0.01	<0.01
Heritability (%)		96.92	95.97	97.77	99.87	95.81	96.64

Significant at the 0.01 probability level.

### Correlations among traits

Table 3 showed that in the four environments, TKW had a significant positive correlation with all kernel traits except KLW. However, the correlations in different environments were unequal. There were significant negative correlations between KLW and KA, KLW and KW. The positive correlations between KA and KP, KL and KW were also significant. The results showed no clear significant correlations between KP and KL, KW, KLW.

**Table 3:** Phenotypic correlations among the six investigated traits.

Env.	Trait	TGW	KA	KP	KLW	KL	KW
E1	TKW	1					
	KA	0.916^**^	1				
	KP	0.684^**^	0.876^**^	1			
	KLW	−0.742^**^	−0.628^**^	−0.188^**^	1		
	KL	0.473^**^	0.674^**^	0.933^**^	0.136^**^	1	
	KW	0.923^**^	0.925^**^	0.641^**^	−0.870^**^	0.359^**^	1
E2	TKW	1					
	KA	0.438^**^	1				
	KP	0.335^**^	0.885^**^	1			
	KLW	−0.233^**^	−0.194^**^	0.128^**^	1		
	KL	0.259^**^	0.785^**^	0.893^**^	0.443^**^	1	
	KW	0.450^**^	0.864^**^	0.623^**^	−0.648^**^	0.387^**^	1
E3	TKW	1					
	KA	0.741^**^	1				
	KP	0.561^**^	0.913^**^	1			
	KLW	−0.391^**^	−0.327^**^	0.012	1		
	KL	0.509^**^	0.807^**^	0.937^**^	0.280^**^	1	
	KW	0.729^**^	0.903^**^	0.694^**^	−0.691^**^	0.494^**^	1
E4	TKW	1					
	KA	0.194^**^	1				
	KP	0.184^**^	0.941^**^	1			
	KLW	−0.325^**^	−0.174^**^	0.104^*^	1		
	KL	0.185^**^	0.841^**^	0.944^**^	0.373^**^	1	
	KW	0.151^**^	0.889^**^	0.726^**^	−0.591^**^	0.523^**^	1

Significance: *P < 0.01; **P < 0.001.

### Linkage map construction

The linkage map in our study consists of 205 SSR markers on all 21 chromosomes. It covers 4672.55 cM of the whole-genome, and the average distance between each mark is 22.79 cM. Chromosome 5D has the lowest number of SSR markers (five) and the shortest length of the genetic maps (221.3 cM). Chromosome 3B has the highest number of SSR markers (15) and the longest length of the genetic maps (408.69 cM).

### QTLs for kernel traits

**Figure 2:**
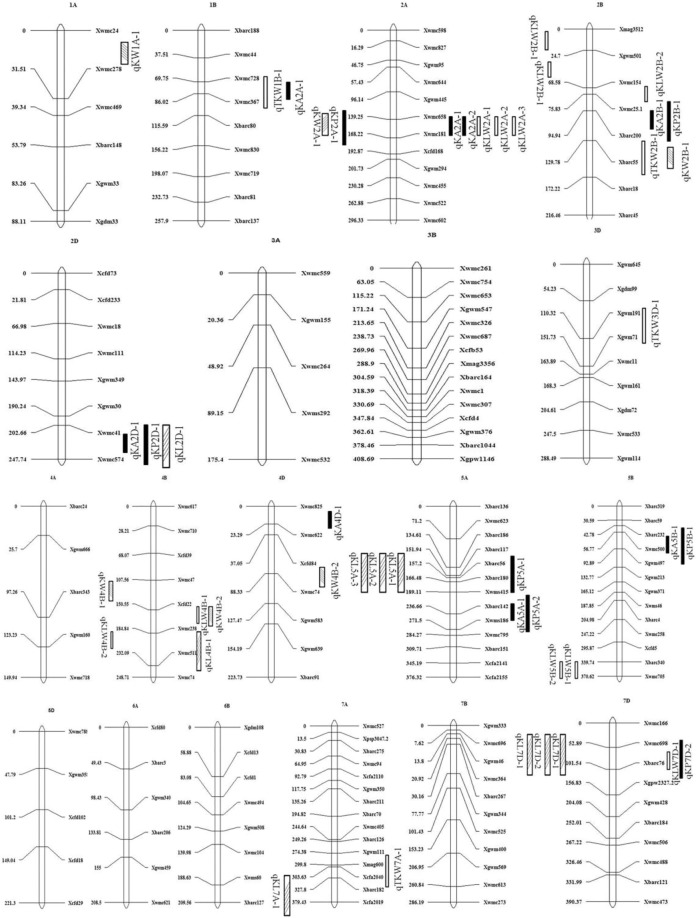
Locations of QTLs for kernel related trait.

Using the Biparental Populations (BIP) module of the inclusive composite interval mapping (ICIM) in the software QTL IciMapping 4.1 for QTL analysis, a total of 39 QTLs for kernel-related traits were found and mapped within nineteen marker intervals on 1A, 2A, 5A, 7A, 1B, 2B, 4B, 5B, 2D, 3D, 4D, and 7D chromosomes (Table 4; Fig. 2), including two for TKW, eight for KA, six for KP, ten for KLW, eight for KL, and five for KW, respectively. Among all the 39 QTLs identified, 13, 5, 5 and 16 were identified in E1, E2, E3 and E4, respectively. Furthermore, the results showed that there are one QTL for KA, one QTL for KLW and two QTLs for KL that were detected in more than one environment, which can be considered stable QTLs.

**Table 4:** QTLs for kernel traits and QTL × environment interaction detected in the Dalibao × BYL8 RIL population.

Trait	Env.	QTL	ICIM					GCIM					Previous QTL
			Marker interval	site	LOD	Add	R^2^ (%)	Marker interval	site	LOD	Add	R^2^ (%)	
TKW (g)	E1	qTKW1B-1						Xwmc728-Xwmc367	76	2.797	−3.742	11.220	*QGw.ccsu-1A, QGw.ccsu-1B, QGw.ccsu-2B*, *QGw.ccsu-5A, QGw.ccsu-6A, QGw.ccsu-6B*, *QGw.ccsu-7A, QGw.ccsu-7D (Mir eral,2010); QTkw.sdau-1D,QTkw.sdau-2A,QTkw.sdau-5D* *(Sun et al.2009);* *QTkw.ncl-2D,QTkw.ncl-4B,QTkw.ncl-5B* *(Ramyaetal,2010);* *QTkw-4A (Cui et al.2015);* *QTgw.aww-3B (Maphosa et al.2014);* *QTKW.ndsu.3D (Kumar et al.2016);*
		qTKW7A-1						Xmag600-Xcfa2040	301	3.1	−3.451	9.544	
	E4	qTKW2B-1	Xbarc200-Xbarc55	104	2.841	2.436	9.454						
		qTKW3D-1	Xgwm191-Xgwm71	146	3.01	−2.38	10.884	Xgwm191-Xgwm71	146	2.624	−2.188	11.578	
KA (mm^2^)	E1	qKA1B-1	Xwmc728-Xwmc367	78	2.847	−0.987	9.731	Xwmc728-Xwmc367	76	2.633	−0.962	10.537	*QGa.ccsu-2A, QGa.ccsu-2B, QGa.ccsu-3B*, *QGa.ccsu-4B, QGa.ccsu-5A, QGa.ccsu-5B*, *QGa.ccsu-6D, QGa.ccsu-7A (Tyagietal.2014);* *QGas.ccsu-5D, QGas.ccsu-7D (Kumari et al.2018);* *QGar.aww-2D, QGar.aww-6A (Maphosa et al. 2014);* *QKA.ndsu.3A,QKA.ndsu.3D,QKA.ndsu.4A*, *QKA.ndsu.4B, QKA.ndsu.5A, QKA.ndsu.5B* *(Kumar et al.2016)*
		qKA2A-1	Xwmc658-Xwmc181	134	2.704	1.505	11.670						
		qKA4D-1	Xwmc622-Xwmc825	0	2.57	−0.827	3.495	Xwmc622-Xwmc825	0	2.845	−0.849	8.207	
	E3	qKA2A-2	Xwmc658-Xwmc181	155	3.416	0.999	13.989	Xwmc658-Xwmc181	155	3.042	1.141	31.332	
		qKA5B-1	Xbarc232-Xbarc500	297	2.68	0.576	4.355						
	E4	qKA2B-1	Xwmc25.1-Xbarc200	150	4.262	0.66	8.183	Xwmc25.1-Xbarc200	150	4.195	0.812	16.559	
		qKA2D-1	Xwmc41-Xwmc574	128	2.635	−0.458	3.894	Xwmc41-Xwmc574	128	3.103	−0.494	6.133	
		qKA5A-1	Xbarc142-Xbarc415	158	4.343	0.906	15.195	Xbarc142-Xbarc415	158	4.294	0.976	23.916	
KP (mm)	E1	qKP5A-1	Xbarc56-Xbarc180	160	2.525	0.293	8.115	Xbarc56-Xbarc180	160	2.559	0.307	9.721	*QGpl.ccsu-4A, QGpl.ccsu-5A,QGpl.ccsu-7B* *(Kumari et al.2018);* *QGp.ccsu-2B, QGp.ccsu-3B, QGp.ccsu-5D*, *QGp.ccsu-7D (Tyagi et al.2014);* *QGp.ccsu-1D, QGp.ccsu-2D, QGp.ccsu-3D (Zhao et al.2015)*
	E2	qKP7D-1						Xbarc76-Xwmc698	137	2.635	0.415	21.517	
	E3	qKP2A-1	Xwmc658-Xwmc181	156	3.106	0.517	12.930	Xwmc658-Xwmc181	156	2.579	0.535	27.718	
		qKP5B-1	Xwmc232-Xbarc500	43	2.695	0.296	3.973						
	E4	qKP2B-1	Xwmc25.1-Xbarc200	86	4.068	0.327	7.977	Xwmc25.1-Xbarc200	86	3.321	0.354	14.476	
		qKP2D-1	Xwmc41-Xwmc574	247	4.457	−0.299	6.613	Xwmc41-Xwmc574	247	4.929	−0.302	10.491	
		qKP5A-2	Xbarc415-Xbarc142	221	4.52	0.455	15.294	Xbarc415-Xbarc142	221	3.757	0.409	19.277	
KLW	E1	qKLW2A-1	Xwmc658-Xwmc181	153	5.11	−0.184	7.020	Xwmc658-Xwmc181	152	3.335	−0.179	18.434	
		qKLW2B-1	Xmag3512-Xgwm501	0	2.596	0.093	1.321						*QGlwr.ccsu-1A, QGlwr.ccsu-2B, QGlwr.ccsu-6A, QGlwr.ccsu-7B (Kumari et al.2018);* *QKw/kl.nwafu-7D,QKw/kl.nwafu-2D (Li et al.2015);* *QKLWR.ndsu.1B, QKLWR.ndsu.3B, QKLWR.ndsu.4A, QKLWR.ndsu.4B, QKLWR.ndsu.5A, QKLWR.ndsu.5B, QKLWR.ndsu.5D, QKLWR.ndsu.7A (Kumar et al.2016);*
		qKLW4B-1	Xcfd22-Xwmc238	62	4.742	0.216	6.465						
		qKLW4B-2	Xwmc238-Xwmc511	30	4.787	0.218	6.759						
	E2	qKLW2A-2	Xwmc658-Xwmc181	153	3.05	−0.176	5.623						
		qKLW5B-1	Xbarc340-Xwmc705	367	3.033	−0.057	8.952						
	E4	qKLW2A-3	Xwmc658-Xwmc181	153	3.36	−0.147	6.261	Xwmc658-Xwmc181	152	3.432	−0.156	28.540	
		qKLW4D−1	Xcfd84-Xwmc74	64	3.435	0.079	7.901						
		qKLW5B-2	Xbarc340-Xwmc705	363	2.849	−0.057	5.542						
		qKLW7D-1	Xbarc76-Xwmc698	100	3.372	0.075	9.538	Xbarc76-Xwmc698	100	3.026	0.071	30.130	
KL (mm)	E1	qKL2B-1	Xwmc501-Xwmc154	57	3.84	0.179	9.948	Xwmc154-Xwmc25.1	72	3.326	0.137	11.111	*QKl.sdau-2B, QKl.sdau-1A, QKl.sdau-1B, QKl.sdau-4A, QKl.sdau-4B (Sun et al.2009); QKl.ncl-5A, QKl.ncl-5B, QKl.ncl-5D (Ramya et al.2010);* *QGl.ccsu-2A, QGl.ccsu-3A, QGl.ccsu-3B, QGl.ccsu-6B, QGl.ccsu-7A, QGl.ccsu-7 (Tyagi et al.2014);* *Q02, Q06, Q07, Q08 (Williams et al.2013);* *QKl.nwafu-6D, QKl.nwafu-7B (Li et al.2015)*
		qKL5A-1	Xbarc56-Xbarc180	162	2.693	0.114	4.006	Xbarc56-Xbarc180	159	2.998	0.125	9.242	
		qKL7D-1	Xbarc76-Xwmc698	134	3.806	0.196	11.817						
	E2	qKL4B-1						Xwmc511-Xwmc238	21	3.615	0.193	21.982	
		qKL5A-2						Xbarc56-Xbarc180	162	2.629	0.103	6.218	
		qKL7D-2	Xbarc76-Xwmc698	137	2.695	0.158	9.685	Xbarc76-Xwmc698	136	3.765	0.182	19.618	
	E3	qKL7D-3						Xbarc76-Xwmc698	148	2.538	0.2	29.101	
	E4	qKL2B-2	Xwmc154-Xwmc25.1	57	3.84	0.179	9.948	Xwmc154-Xwmc25.1	72	3.326	0.137	11.111	
		qKL2D-1	Xwmc41-Xwmc574	135	4.077	-0.145	13.222						
		qKL5A-3	Xbarc56-Xbarc180	162	2.693	0.114	4.006	Xbarc56-Xbarc180	159	2.998	0.125	9.242	
		qKL7A-1	Xbarc182-Xcfa2019	379	2.637	-0.089	4.843						
KW (mm)	E1	qKW4B-1	Xwmc47-Xcfd22	105	2.777	-0.223	6.094						*QKw.sdau-5D, QKw.sdau-6A, QKw.sdau-2A* *(Sun et al.2009);* *QKw.ncl-1D, QKw.ncl-2B, QKw.ncl-2D, QKw.ncl-4B, QKw.ncl-5B (Ramya et al.2010);* *QGwid.ccsu-6B,QGwid.ccsu-6D,QGwid.ccsu-3B*, *QGwid.ccsu-7D (Tyagi et al.2014);* *Q04 (Willians et al.2013);* *QKw.nwafu-6D, QKw.nwafu-7B, QKw.nwafu-4D, QKw.nwafu-1A (Li et al.2015);* *QKW.ndsu.7A (Kumar et al.2016);*
		qKW4B-2	Xcfd22-Xwmc238	61	2.708	−0.193	4.508						
	E2	qKW1A-1	Xwmc24-Xwmc278	110	2.753	0.088	7.774	Xwmc24-Xwmc278	23	2.934	0.079	15.367	
	E3	qKW2A-1	Xwmc658-Xwmc181	135	2.556	0.112	9.145	Xwmc658-Xwmc181	155	2.797	0.134	29.455	
	E4	qKW2B-1	Xbarc200-Xbarc55	103	2.605	0.076	8.325						

E1: Yangling 2016–2017, E2: Yangling 2017- 2018, E3: Nanyang 2017–2018, E4: Suqian 2017–2018.

LOD: Maximum-likelihood LOD score for the QTL calculated by IciMapping 4.1.

Add: ± Additive effect. Positive values indicate a positive effect of Dalibao alleles, whereas negative values indicate the contribution of the BYL8 allele.

PVE (%) = phenotypic variance estimated from marker regression against phenotype.

AbyE: QTL × environment interaction.

Genome-wide composite interval mapping (GCIM) method was also used to conduct QTL analysis. A total of 28 QTLs were distributed on chromosomes 1A, 2A, 5A, 7A, 1B, 2B, 4B, 2D, 3D, 4D, and 7D, including three for TKW, six for KA, six for KP, three for KLW, eight for KL, and two for KW, respectively. Out of the 28 identified QTLs, including 8, 5, 4 and 11 QTLs in E1, E2, E3, and E4, respectively.

Among all the QTLs detected by the ICIM and GCIM methods, there were 22 common QTLs, and six additional QTLs were also identified by the GCIM method. Among these 6 QTLs, including 3, 2, and 1 QTLs for KL, TKW, and KP had been detected, respectively. Therefore, we combined the results of these two modules and identified 45 QTLs for six kernel traits. The 45 QTLs contributed to 12 chromosomes. A total of 4, 8, 17, 11, and 5 QTLs were identified for TKW, KA, KP, KLW, KL, and KP, respectively. Among the 45 QTLs, we performed stepwise regression analysis of all the QTLs on each trait. And then we introduced in details all the QTLs remained in the best regression equation of the stepwise regression analysis. At this time, the number of significant QTLs is 21.

Among the above 21 significant QTLs, two were found to be associated with TKW. One QTL (*qTKW3D-1*) was identified commonly by the ICIM and GCIM methods and located between markers *Xgwm191* and *Xgwm71* on chromosome 3D. Another was located between markers *Xwmc728* and *Xwmc367* on chromosome 1B. Each of the two QTLs accounted for approximately 11.0% of the PV in E4 (2017–2018, Suqian) and E1 (2016–2017, Yangling). Their novel alleles were derived from BYL8.

Five QTLs for KA were considered as significant QTLs, located on chromosomes 1B, 2A, 2B, 2D, 4D, and 5A. One QTL (*qKA2A-2*) was identified by only ICIM model and located between marker *Xwmc658* and *Xwmc181* on chromosome 2A, explained 11.670% of the PV in E3 (2017–2018, Nanyang), with positive effects coming from Dlibao. Moreover, the novel allele of *qKA2B-1*and *qKA5A-1* were also derived from Dalibao, they were located within respective marker intervals *Xwmc25.1-Xbarc200* and *Xbarc142-Xbarc415*, explained 8.183%–23.916% in E4. Another two QTL, *qKA1B-1* and *qKA4D-1* was within marker interval *Xwmc728-Xwmc267* and *Xwmc622-Xwmc825*, accounted for 3.495% –10.537% of the PV in E1. Their novel alleles were derived from BYL8.

Three QTLs associated with KP were mapped onto chromosomes 2A, and 5A, explaining 8.115%–27.718% of PV. They were identified commonly by the ICIM and GCIM methods and their novel alleles were derived from Dalibao. The QTL, *qKP5A-1* was located between marker *Xbarc56* and *Xbarc180* in E1. Another two QTLs, *qKP2A-1* and *qKP5B-1* were within marker intervals *Xwmc658-Xwmc181* and *Xbarc232-Xbarc500* in E3.

Four significant QTLs for KLW were mapped onto chromosomes 2A, 5B, and 7D. In particular, two QTLs (*qKLW2A-1*and *qKLW2A-3*) were detected in the marker interval between *Xwmc658* and *Xwmc181* on chromosome 2A in two different environments, explaining 6.262%–28.540% of PV, this locus can be considered as a major QTL for KLW. They were identified commonly by the ICIM and GCIM methods and their novel alleles were derived from BYL8. One QTL, *qKLW5B-1* within the marker interval between *Xbarc340* and *Xwmc705* detected in E2 (2017–2018, Yangling), with positive effects coming from also BYL8. And one QTL, *qKLW7D-1*, was within the marker intervals between *Xbrac76* and *Xwmc698* in E4. It was identified commonly by the ICIM and GCIM methods and novel allele was derived from Dalibao.

Five significant QTLs associated with KL were detected on chromosomes 2B, 2D, and 7D, explaining 9.685–19.618% of the PV. Of these QTLs, two QTLs (*qKL7D-1*and *qKL7D-2*) were detected in the marker interval between *Xbrac76* and *Xwmc698* on chromosome 7D in two different environments. Their novel allele was derived from Dalibao but only *qKL7D-2* was identified commonly by ICIM and GCIM methods. Another two QTLs (*qKL2B-1* and *qKL2B-2*) were located within marker interval *Xwmc501-Xwmc154* and *Xwmc154- Xwmc25.1* on the chromosome 2B detected in E1 and E4, respectively. They were identified commonly by ICIM and GCIM methods and their novel allele were derived from Dalibao. Another QTL, *qKL2D-1* in the marker intervals between *Xwmc154* and *Xwmc25.1*detected in E4, had positive effects from BYL8.

Two QTLs (*qKW1A-1* and *qKW2A-1*) for KW were considered as significant QTL. They were identified commonly by the ICIM and GCIM methods and located within markers intervals *Xwmc24-Xwmc278* and *Xwmc658-Xwmc181*, accounted for 15.367% and 29.455% of the PV in E2 and E3. Their novel alleles were derived from BYL8.

### Pleiotropic QTLs for kernel traits

QTLs related to different kernel traits could be found in the same marker intervals, possibly due to the pleiotropic effect of a single gene or a set of tightly linked genes. In this study, four QTL clusters related to KA, KP, KLW, KL, and KW kernel traits were located on chromosomes 2A, 2B 5A, and 7D (Table 5). A QTL cluster for KA on chromosome 2A was found in two environments, while other QTL clusters were detected in one environment.

**Table 5:** Co-localized QTLs for different kernel traits in wheat.

Chromosome	Kernel traits	Marker interval	LOD score	H^2^
2A	KLW,KA,KP and KW	Xwmc658-Xwmc181	2.556–5.110	6.261–29.455
2B	KA and KL	Xbarc200-Xbarc55	2.605–2.841	8.183–11.111
5A	KA and KP	Xbarc56-Xbarc180	2.525–4.523	8.115–23.916
7D	KLW and KL	Xbarc76-Xwmc698	2.538–3.765	9.538–30.130

*TKW* thousand kernel weight, *KA* kernel area, *KP* kernel perimeter, *KLW* kernel length/width ratio, *KL* kernel length, *KW* kernel width.

### BSA+ 660 K result analysis

**Figure 3:**
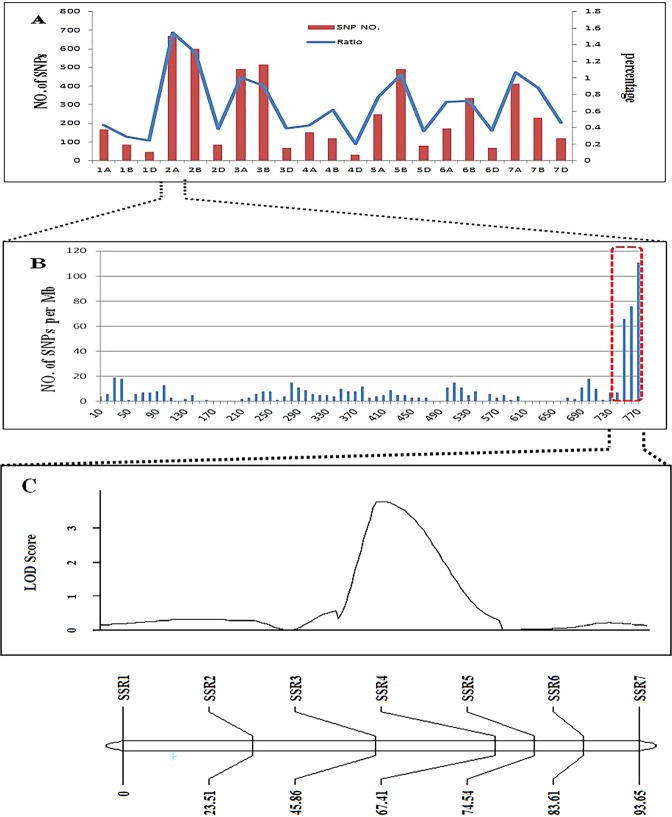
*A* Distribution of polymorphic SNPs in each chromosome identified by the 660 K SNP array and corresponding percentages. *B* Positions of SNPs in chromosome 2A based on the 660 K SNP. *C* LOD Score and genetic linkage map.

After genotyping with 660 K SNP array, the number of homozygous polymorphisms SNP between DNA bulks and their parental lines were counted, resulting in a total of 5285 SNPs. The chromosome 2A had the highest number of SNPs (670). The others were located on the rest of chromosomes (Fig. 3). Furthermore, for chromosome 2A, the proportion of SNPs that overlapped between the bulk and the parent was the highest. The numbers and the proportions of SNPs on chromosomes 5B, 7A, 3A, and 4B were also higher. These results indicated that SNPs in 2A were extremely likely to be associated with the major locus and there may be minor QTLs on 5B, 7A, 3A, and 4B. Most of the SNPs on 2A were within the interval *Ax-94815470–Ax-109351630* spanning 28.8 Mb *(2*A*:751004612–2*A*:779844131)* in the 660 K map. We employed a BLAST search to obtain physical positions of polymorphic SSR markers using the SSR forward primer, which is the newly released Chinese Spring sequence (Reference Sequence v1.0, the International Wheat Genome Consortium (IWGSC), http://www.wheatgenome.org/). The markers *Xwmc658* and *Xwmc181* spanned 42.6 Mb *(2*A*:728609541–2*A*:771166682)*. The QTL mapped for KLW was located between the SSR markers *Xwmc658* and *Xwmc181*, with a genetic distance of 28.9 cM, corresponding to a physical interval of 42.6 Mb in the Chinese Spring RefSeq v1.0 sequence. By contrast, this physical location contains the segment of most of the SNPs in the 660 K results. BSA combined with the 660 K gene chip has located a major QTL for KLW in a smaller region. Subsequently, a genetic map constructed using the 7 newly developed SSR markers (Table 6) and data from the 547 F_7_ RILs spanned 93.65 cM. A major QTL for KLW located in a 21.55 cM interval spanned by SSR3 and SSR4 corresponding to a physical interval of 10.8 Mb in the Chinese Spring RefSeq v1.0 sequence.

**Table 6:** Primer sequence for new developed SSR marker.

Marker	Tm	GC%	Forward primer	Reverse primer
SSR1	56.4	50	CGACAACGACATACCCGATACA	AGAAGAGGAGAGGAAGAAGAGG
SSR2	58.4	57.1	TCCCAACAAACACACCGCAAA	CGATCCCGTCTGGATGCCTTT
SSR3	56.8	54.5	TGAAACCTATGGCCCACGG	GCAAGTCACCTCGTACCTACAC
SSR4	55.7	45.5	AAGAGAAACCTTCAGCGAGACA	GCCAGAGCCGGACTTGATTC
SSR5	56.1	52.4	CAGTTGTCTCACCCTCGTTGT	CGTAGGCTGCTTGCTCATCC
SSR6	57.8	54.5	CCGTTTGGACCACGTACCTAGA	ACCTACTTGAGGGCACCGAATG
SSR7	58.1	50	CGCCGAACAAACTCACCATTCA	AGTAGCACGCTTGGCACCA

## Discussion

### QTLs for kernel traits

The present study investigated six kernel traits using a RIL population consisting of 142 individuals from the cross Dalibao/BYL8. Plant materials were grown under four environmental conditions. A total of 45 QTLs were identified for six kernel-related traits on the chromosomes 1A, 2A, 5A, 7A, 1B, 2B, 4B, 5B, 2D, 3D, 4D, and 7D across four environments. Among these 45 QTLs, there were 21 QTLs were considered as significant QTLs for six kernel traits.

TKW plays an important role in increasing wheat yield. In our study, significant QTLs for TKW were mapped onto the chromosome 1B and 3D. Previous studies have also found QTLs for TKW on the chromosome 3D^6,17,24,25^. Earlier studies also found QTLs related to kernel weight on the 3D chromosome in synthetic wheat^26^. In addition, there are many reports of QTLs for TKW on chromosome 1B^1,7,27,28^. However, previous studies have found QTLs for TKW on almost all chromosomes of the wheat genome^1,2,6–8,10–13,25,27–35^. Kernel-related traits are susceptible to environmental impact, and the observed values of the same plant materials in different environments were also significantly different.

For KA, significant QTLs were located on chromosomes 1B, 2A, 2B, 4D, and 5A. Campbell^1^, Kumar^6^, Mir ^7^ and Tyagi^12^ also detected QTLs associated with KA on the chromosome 2A. Campbell^1^, Tyagi^12^ and Maphosa^16^detected a QTL on 2B. Breseghello^5^, Kumar^6^ and Tyagi^12^ detected QTLs on 5A. QTL *(qKA4D-1)* and QTL *(qKA1B-1)* were found at a new location. Other earlier studies have reported QTLs for KA on the chromosomes 1A, 3A, 4A, 6A, 7A, 3B, 4B, 1D, 3D, 5D, 6D, and 7D^1,6,10,12,15–17^.

QTLs for KP were previously reported on chromosomes 1A, 1D, 2B, 2D, 3B, 3D, 4A, 4D, 5A, 5D, 6B, 6D, 7B, and 7D^1,10,12,15,17^. In the present study, QTLs for KP were detected on chromosomes 2A, and 5A.We also found QTLs on chromosomes 2A that have not been reported previously.

At present, research on the KLW of wheat is not deep enough, previous studies found QTLs on chromosomes 1A, 1B, 2B, 2D, 3B, 4A, 4B, 5A, 5B, 5D, 6A, 7A, 7B, and 7D^6,14,15^. In our study, significant QTLs for KLW were also detected on chromosomes 2A, 5B and 7D. QTL *(qKLW2A-1)* was found in a new chromosome.

With respect to KL, our study detected five QTLs on chromosomes 2B, 2D, and 7D in three environments. Many previous studies have found QTLs for KL on 2B and 2D^1,2,5,8,11–14,16^, indicating that these two chromosomes have an important influence on wheat kernel traits. Okamoto^10^ detected a QTL for KL on chromosome 7D.

Significant QTLs for KW on chromosome 1A and 2A were detected in this study. Campbell^1^ and Li^14^ detected QTLs for TW located on chromosome 1A. In addition, there are many reports of QTLs for KW on chromosome 2A^1,8,12,13^. Among these, Sun^8^ detected a QTL associated with marker *Xwmc181* on chromosome 2A, the same marker found in our study. Previous studies have also found QTLs on all of the other chromosomes (except for 3A), for which none were found in our study^2,5,6,10,15–17,27,36,37^.

We detected some consistent QTLs for TKW, KL, KW, KA, KP, and KLW in various environments. It is believed that these QTLs can be used for marker-assisted selection breeding and fine mapping.

### Pleiotropic QTLs for kernel traits

Among QTLs for kernel traits detected in this study, 4 regions controlled two or more kernel traits at the same time forming overlapping QTLs. There is a significant correlation between kernel traits at the QTL level. In earlier studies, some pleiotropic QTLs related to kernel traits were reported^8,11,13–15,28,35,36,38–40^. In this study, there were seven pleiotropic QTLs related to KA, KP, KLW, KL, and KW located on chromosomes 2A, 2B, 5A, and 7D.

Five QTLs, one each for KLW *(qKLW2A-1)*, KA *(qKA2A-2)*, KP *(qKP2A-1)* and KW *(qKW2A-1)* were located on chromosome 2A within the marker interval between *Xwmc658* and *Xwmc181*. The marker *Xwmc181* closest to this pleiotropic QTL was earlier reported by Sun^8^ for KW and TKW. Kumari^15^ detected three pleiotropic QTLs for KLW and KW, KLW and KL, and KW and KA on chromosomes 6A, 7B, and 7D. The pleiotropic QTL on the 2A chromosome associated with KA was found in two environments, while QTLs for KLW, KP and KW were found in one environment. The QTL effects between KA, KP, KW and KLW were negative, and the relationships between KA, KP and KW were positive, in accordance with the simple correlation analysis results.

Two QTLs, one each for KA *(qKA2B-1)* and KL *(qKL2B-2)* were co-localized on chromosome 2B in the marker interval between *Xbarc200* and *Xbarc55*. Each of these QTLs was detected in only one environment. There was positive relationship between these three traits, consistent with the simple correlation analysis results.

Two QTLs, one each for KA *(qKA5A-1)* and KP *(qKP5A-1)*, were mapped onto chromosome 5A within the marker interval between *Xbarc56* and *Xbarc180*. Each of these QTLs was detected in only one environment. The parental genotype Dalibao displayed positive effects for the above QTLs, and the relationship between these two traits was consistent with the results of the simple correlation analysis.

A QTL each for KLW *(qKLW7D-1)* and KL *(qKL7D-1)* were mapped in the marker interval between *Xbarc76* and *Xwmc698* on chromosome 7D. Each of these QTLs was detected in only one environment. The positive relationships between the three traits were in agreement with the simple correlation analysis results. Ramya^11^ previously found a pleiotropic QTL for KLW and KL on chromosome 4B. Li^14^ have detected a pleiotropic QTL for KLW and KL on chromosomes 1A and 2D.

In this study, QTLs for KL and KW were detected on chromosomes 2B, 2D, 7D and 1A, 2A, respectively. There were no pleiotropic QTLs between these two traits, but the correlation between KL and KW was high. This is not consistent with previous research results that KL and KW were largely under independent genetic control^41^. As for TKW, the correlations between TKW and KL, KW, KLW, KA and KP were significant. Campbell^1^ previously reported that KL and KW influenced the QTL for TKW, but no pleiotropic QTLs were found in our study. Previous studies on KLW traits are relatively rare, so no results similar to ours are available for comparison.

These common or overlapping QTL regions need to be fine mapped to determine whether the regions are pleiotropic in different QTLs or are closely linked QTLs that control individual traits.

### Comparison of BSA+ 660 K and SSR results

In our study, we combined BSA with the 660 K SNP array to search for major QTLs for KLW that have not been reported previously. The results suggest that the major QTLs were extremely likely to be related with the SNPs on chromosome 2A, and there may be minor QTLs on 5B, 7A, 3A, and 4B. These results can be verified using SSR markers. However, no genetic positions in relation to the wheat 660 K SNPs have been documented^35^. We can use SSR marker forward primers and SNP probes to obtain physical locations by BLAST using the Chinese Spring RefSeq v1.0 sequence. In subsequent work, we developed more SSR markers based on the results from the wheat 660 K to validate the accuracy. These results should be valuable for the wheat breeder to improve the kernel traits via marker-assisted selection breeding.

## Materials and Methods

### Plant materials

The plant materials used in this experiment were from a recombinant inbred line (RIL) population at the F_7_ generation consisting of 547 lines produced from a cross between Dalibao and BYL8. Dalibao has a large kernel length-width ratio (KWL = 2.801), and BYL8 has a small kernel length-width ratio (KWL = 2.167). These 547 lines and their parents were grown from 2016 to 2017 in Yangling, Shaanxi Province, China (E1), and from 2017 to 2018 in Yangling, Shaanxi Province, China (E2), Nanyang, Henan Province, China (E3) and Suqian, Jiangsu Province, China (E4). The plant materials were sown on October 2, 2016 (E1), September 30, 2017 (E2), November 7, 2017 (E3) and October 6, 2017 (E4). All four environments consisted of 549 rows, each 1 m long and spaced 30 cm apart, with 10 plants in each row. There were no differences in field management arising from local agricultural practice.

### Phenotype analysis

At maturity, wheat plants were bulk-harvested and sun-dried for phenotypic evaluation. Three individuals from each family were randomly selected and threshed separately. Then, at least 200 fully filled kernels of each line were used to measure six kernel-related traits, (KL, KW, KP, KA, KLW and TKW) using the Wanshen kernel testing equipment developed by Wanshen Science and Technology Ltd. (Hangzhou, China; http://hzwseen.foodmate.net/). All the above traits from the four environments were measured as the average of each family from three replicates.

### Statistical analysis

The calculations of descriptive statistics, Student’s t-test, correlation analysis, and analysis of variance (ANOVA) were performed using SPSS 21.0 software (http://www.spss.com). The phenotypes of six kernel traits used for statistical analysis in the four environments were from the averages of three replicates per family. Additionally, the GLM model in SPSS was used to compute the broad-sense heritability (*h*^2^). Frequency distributions for six kernel traits were drawn using the Microsoft Office Excel 2007 software (https://www.microsoft.com).

### Genotype analysis and QTL mapping

Genomic DNA was extracted from young leaves of both parents and families by the hexadecyl trimethyl ammonium bromide (CTAB) method. TE buffer was used to dissolve extracted DNA, and the DNA quality was detected by 1% agarose electrophoresis. DNA was stored at −20 °C. A total of 142 individuals were genotyped using 205 SSR markers. These 205 SSR markers were evenly selected from the 21 chromosomes of the whole genome using the GrainGenes database (http://wheat.pw.usda.gov/GG2). A 6% denaturing polyacrylamide gel electrophoresis (PAGE) was used to separate PCR conducts. Using the Biparental Populations (BIP) module of the inclusive composite interval mapping (ICIM) in the software QTL IciMapping 4.1 (http://www.isbreeding.net) for QTL analysis^42^. The critical LOD scores for a significant QTL were set at 3.0. The GCIM method in the software QTL.gCIMapping from the R website (https://cran.r-project.org/web/packages/QTL.gCIMapping/index.html) was also used to identify QTLs for the above traits, with the purpose of identifying the results from the ICIM method^43,44^; the critical LOD scores for a significant QTL was also set at 3.0, and the walking speed for the genome-wide scan was set at 1 cM.

### SNP Genotyping

Based on the phenotypic investigation, we used BSA to identify polymorphic markers between the small kernel length-width ratio and large kernel length-width ratio parents, and two extreme pools (KLW < 1.967 and KLW > 2.608). Two bulks were composed of equal amounts of DNA from 25 lines with low KLW and 25 with high KLW. Genotyping of the two F_7_ bulks and the parents was performed using the 660 K SNP arrays from CapitalBio Corporation (Beijing, China; http://www.capitalbio.com).

### Development of Wheat SSR Markers on 2A Chromosome

The sequence of the target segment from the whole genome of wheat was obtained, and SSR Hunter software was used to find three types of SSRs of two, three, and four nucleotides. The recognition criteria are as follows: the length of the repeat sequence was ≥60 bp, that is, the number of repetitions was greater than or equal to 30, 20, and 6, respectively. Primers were designed based on the flanking regions of the SSR using Primer 5.0 software. The main parameters of the primer design are as follows: the start and end positions of the SSR sequence were not less than 20 bp from the 5′ and 3′ ends, respectively; the primer length was 18 ~ 25 bp; the primer GC content was 40–60%; the annealing temperature Tm value was 50–65 °C, and the Tm values of the forward and reverse primers did not exceed 5 °C; the length of the PCR amplification product was 100–400 bp; and the score was 80 or higher.
